# Advanced Method for the Detection of Saturated Monoglycerides in Biodiesel Using GC-EI-MS/MS

**DOI:** 10.1021/acsomega.4c00513

**Published:** 2024-05-24

**Authors:** Jessica Pichler, Marcella Frauscher, Martina Marchetti-Deschmann

**Affiliations:** † AC2T research GmbH, Viktor-Kaplan-Straße 2C, 2700 Wiener Neustadt, Austria; ‡ Institute of Chemical Technologies and Analytics, TU Wien, Getreidemarkt 9/164, 1060 Vienna, Austria

## Abstract

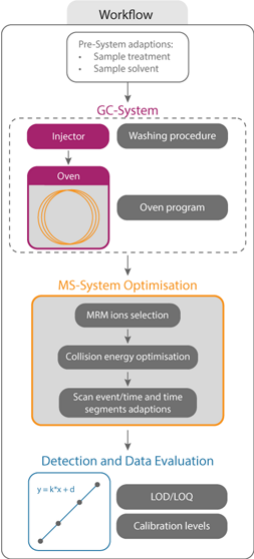

Common B7 biodiesels consist of mixtures of mineral oil-based diesel and 7% fatty acid methyl ester (FAME). While biocontent increase can be achieved with these blends at high-quality levels during cold temperature periods, fuel filter blocking events are reported from time to time. Based on a preliminary study on fuel filters, the selection of compounds responsible for filter blocking could be narrowed down to saturated monoglycerides (SMGs). The most abundant SMGs in Europe are 1- and 2-monopalmitin (1-C16:0, 2-C16:0) and 1-monostearin (C18:0), based on the FAME origin. Until now, there has been no simple, precise, and accurate method to quantitatively detect those SMGs in the B7 matrix, which was the aim of the following work. An improved gas chromatography electron ionization tandem mass spectrometry method was developed for the quantitative detection of 1-C16:0, 2-C16:0, C18:0, and C20:0 SMGs. During the method improvement, (a) the sample preparation and (b) the calibration were optimized for low concentrations. (c) The samples were analyzed by multiple reaction monitoring focusing on specific qualifier and quantifier ions with optimized collision energies, (d) time segments and improved scan time were implemented, and (e) limits of detection and limits of quantification were determined. The time-stability of SMG standards in CHCl_3_ with 4% neat biodiesel and the discrimination effects of the standard components were evaluated to assess method reliability. Overall, a highly sensitive and precise method for the improved detection of SMGs in biodiesel is presented.

## 1 Introduction

Biodiesel (B100 or FAME) is a fuel produced from renewable resources (e.g., rapeseed, soybean, palm, corn, etc.) by transesterifying their natural oils and fats with methanol. This converts triglycerides into FAME products, with the intermediates mono- and diglycerides still being found in the final biodiesel, affecting its quality.^1^ For passenger cars in Europe, the content of biodiesel is restricted to a maximum of 7 wt % in neat diesel (B0), resulting in the so-called B7 mixtures.^2^

The origin of FAME can be edible feedstock (1st generation), nonedible feedstock (2nd generation), algae feedstock (3rd generation), or microbial feedstock (4th generation). The FAME feedstock used is dependent on the geographical region^3^ and mainly comprises first-generation crop oils derived from palm, rapeseed/canola, soybean, corn, and sunflower; used cooking oils (UCO); and animal fats.^4,5^ The primary feedstocks in Europe are rapeseed and palm oil, UCO, and minor amounts of soybean oil, sunflower oil, and animal fats.^6^ In the US, mostly soybean, corn, and canola oils are used, as well as recycled feedstocks.^7^ A FAME’s fatty acid profile depends on the FAME feedstock type used,^8^ influencing, for example, the cold flow properties of the fuel.^9^

Unbeneficial cold flow properties of fuel can lead to the blocking of fuel filters, especially with seasonal changes. Biodiesel-related fuel filter blocking incidents have been reported recently in agricultural machinery^10^ and trains,^11^ although only blended with 7% mixtures in diesel. As filter blocking incidents accumulate, investigations of correlations between properties of diesel, biodiesel, and blends (including different FAME feedstocks) causing filter blocking (reduced cold flow properties, particulate matter formation, and oxidation products)^12^ as well as new approaches for the analysis of fuels and filters increase, e.g., for the prediction of cold flow properties of biofuels in relation to their ester profile^13^ or filter analysis with thermal desorption GC-MS providing a simple and fast method, where blocked filters showed different FAME species (mainly C16:0) and glycerol.^14^

Based on preceding studies of numerous fuel filters from all over Austria, the cause of fuel filter blocking could be narrowed down to the occurrence of SMGs, which may precipitate, i.e., at lower temperatures, attaching to the fuel filters and subsequently blocking the fuel passage. Comparing blocked filters to reference filters, high amounts of SMGs from C16:0 to C22:0 were only found on blocked filters, whereas reference filters predominately contained unsaturated C16 to C18 monoglycerides (MGs).^15^

To this point, a maximum content of 0.7 wt % (7000 ppm) total SMGs in biodiesel is allowed by DIN EN 14214:2012+A2:2019,^16^ resulting in 490 ppm of SMG content in ready blended B7. Even though the maximum allowed SMG amount was reduced over the years, the current levels still seem to cause unforeseeable blocking events under certain conditions. Furthermore, the amounts of individual SMG components are not regulated; the limit is given as a sum parameter of all SMGs. By EN 17057:2018-03,^17^ SMGs (as a sum of single contents of 1-C16:0, 2-C16:0, and 1-C18:0) in biodiesel can be detected reliably within the range of 200 to 1500 ppm. The method is suitable for FAME derived from rapeseed, palm, and used cooking oil, but not from palm kernel or coconut derivates (applies for fuel produced and used in Europe).

When diesel needs to be stored for extended periods, e.g., at industrially used fuel stations, for storage at power security generators, or to bridge times of general constraints, in all cases, maintaining the quality of the fuel must be ensured, and therefore, studying the changes in filterability during long-term fuel storage is essential. Moreover, different storage factors (tank design/geometry, filling/extraction systems, etc.) and environmental influences during delivery and storage (mild/harsh climate, season, and temperature changes) should be considered. When storing biodiesel/diesel blends at different temperatures for 12 months, the water content significantly increased and so did the content of mono- and diglycerides and total glycerin. It also showed that the amount of biodiesel mixed with diesel impacts the filterability, especially at lower temperatures. Diesel is much more nonpolar than biodiesel, causing a reduced solubility for polar residues. This leads to more precipitates, an effect that further increases with decreasing temperature. Characterization of such precipitates by GC-FID showed a composition of mainly free glycerin and monoglycerides, especially monopalmitin and monostearin.^18^ Neat B100 can tolerate more SMGs until a critical value is reached, increasing the cloud point (CP). The CP is the temperature below which a transparent solution undergoes a liquid–solid phase transition to form either a stable solution or a suspension that settles a precipitate, besides other effects. The SMG-to-B100 ratio is an essential factor for the CP. Additionally, the polymorphism of SMG crystals with different melting temperatures, solubilities, and stabilities has to be considered. SMG crystallization can occur upon rapid cooling, slow warming, or during storage and can cause fuel filter blocking above the CP.^19^

MGs are determined by GC-FID according to DIN EN 14105^20^ as low as 0.001 wt % (mono-, di-, and triglycerides) along with free glycerol at 0.1 wt % in FAME from oil seeds, animal- and plant-derived fats, oils, and their residues. This method detects 1- and 2-C16:0, 1-C18:0, and 1- and 2-C18:1 to C18:3, while 1-C19:0 is used as the internal standard. In the sense of a B7 mixture, this would result in an LOQ of 70 ppm for free glycerol and 0.7 ppm for total glycerides. When analyzing different types of biodiesels for their MG content by ASTM D6584-17 using GC-FID, it was found that the method needed to be improved for nonconventional feedstocks. Improvements to the method, without changing the analysis procedure, such as using an MG stock standard from C10 to C22, adapting the relative retention time windows, and matching the profiles of MGs and FAME, led to a more accurate determination of monoglycerides.^21^

Despite filter blocking events still occurring within regulatory limits, the risk potential remains unclear for specific fuels. Both the fuel manufacturing industry and distributors are urged to evaluate the filter blocking potential as a quality assurance measure. Typically, multiple analysis methods are employed to evaluate quality criteria across various fuel matrices such as diesel, biodiesel, and blends. However, this approach can be inadequate for industries due to the use of disparate instrumentation and configurations for different matrices, leading to increased resource consumption and costs. Consequently, industrial stakeholders expressed the necessity for a fast and straightforward method to assess the filter blocking potential of petroleum- and bio-based fuels, as well as blends.

Eventually, all named complications led to the requirement of a more advanced detection method using GC-EI-MS/MS for single SMG amounts, being responsible for filter blocking events, and their limits in B0, B7, and B100 matrices, where no published method exists, providing the aim of the presented research work.

## 2 Materials and Methods

### 2.1 Chemicals, Reagents, and Consumables

For method development of monoglyceride identification in biodiesel, the following reference materials were used: 1-monopalmitin (1-C16:0; >99%) [CAS: 542-44-9] obtained from Sigma-Aldrich (St. Louis, MO, USA) and 2-monopalmitin (2-C16:0; >98%) [CAS: 23470–00–0], monostearin (C18:0; 99%) [CAS: 123-94-4], and monoarachidin (C20:0; >99%) [CAS: 30208-87-8] all obtained from Larodan (Solna, Sweden). As internal standard (IS) rac-glycerol 1-myristate (C14:1; >99%) [CAS: 488862-82-4] obtained from Avanti Polar Lipids (Alabaster, AL, USA) was chosen, it is not naturally found in biodiesel in Europe (dependent on the FAME feedstock used). If C14:1 is present in the used biodiesel matrix, the method must be adapted by replacing it with an equivalent IS. A multicomponent standard mix was produced in different concentrations from these reference materials to set up a calibration curve.

BSTFA+TMCS (99:1; *N*,*O*-bis(trimethylsilyl)trifluoroacetamide with trimethylchlorosilane; 99%) [CAS: 25561-30-2] obtained from Sigma-Aldrich (Buchs, Switzerland) was used as a derivatization agent. Following solvents were applied: DCM (≥99.8%) [CAS: 75-09-2] obtained from Sigma-Aldrich (St. Louis, MO, USA), CHCl_3_ (≥99.9%) [CAS: 67-66-3] purchased from Supelco (Burlington, MA, USA), methanol (MeOH; 99%) [CAS: 67-56-1] purchased from Supelco (Darmstadt, Germany), and *n*-hexane (≥95%) [CAS: 110-54-3] obtained from Carl Roth (Karlsruhe, Germany).

### 2.2 Field Sample Collection and Preparation

Different petrol station distributors all over Austria supplied field samples (diesel and biodiesel) throughout changing seasons (varying product composition). B0, B7, and B100 samples were ultrasonicated for 15 min at room temperature and vortexed upon arrival in their original metal container to solubilize any potential precipitates. They were refilled in transparent bottles and stored at 4 °C until usage.

For GC-MS analyses, 1 mL of sample was prepared in 2 mL amber 51 vials with PFTE/silicone crimp seal (both Phenomenex; Torrance, CA, USA), diluted in the solvent, and silylated with BSTFA+TMCS in mass excess (1:4.5) at 70 °C for 1 h. Field samples (B0, B7, and B100) were added to the solvent in a 4 wt % dilution (see Section 3.1 Calibration and Sample Solvent).

For determination of the water content, field samples were measured according to DIN 51777-2 (indirect method)^22^ by Karl Fischer titration, where the sample is heated to 120 °C, water is evaporated, collected in a titration cell, and determined iodometrically.

### 2.3 Calibration Sample Preparation

Method used as the starting point (see Section 2.4 GC-EI-MS method): The calibration samples for detection of SMGs in the B7 field sample matrix contained 1-C16:0, 2-C16:0, C18:0, and C20:0 as a multicomponent mix in the following concentrations: 10, 50, 100, 250, 500, and 1000 ppm, plus 200 ppm of C14:1 as the internal standard (IS), in DCM. Field samples (B0, B7) were prepared as 4 wt % dilutions in DCM.

Improvement: Based on prior analyses,^15^ low or very low amounts of SMGs were expected in the B7 sample matrix. The maximum total SMG content is approximately 500 ppm, which is diluted to approximately 20 ppm after sample preparation in the GC vial. For this, the concentration range had to be adapted to lower LOQs: 1, 2.5, 5, 15, 25, 50, 75, and 100 ppm of reference SMG dilutions with 100 ppm IS were prepared in CHCl_3_ and CHCl_3_ + 4 wt % B0 for evaluation of matrix effects and higher sensitivity. The calibration was adapted for lower or higher levels, depending on the expected SMG concentration.

### 2.4 GC-EI-MS Method

The initial method started with the GC oven preset to 60 °C, and after keeping the temperature constant for one min, it was heated to 300 °C (rate 10 °C/min) and held for 15 min (total run time 40 min). The EI ion source was operated at an electron emission current of 50 μA, and derivatized analytes were detected within the range of *m*/*z* 40 to 650. Monoglyceride precursor ions of *m*/*z* 341 (C14:1), 371 (1-C16:0), 218 (2-C16:0), 399 (C18:0), and 427 (C20:0) were chosen for selected ion monitoring (SIM) in separate scan events with a scan time of one s and 10 V collision energy. A full scan was conducted over the entire run time with a scan time of 0.2 s. Structural identification was done with the MS spectrum, whereas quantification was performed with the FID chromatogram. Sample solvents, washing solvents, and washing procedures were improved throughout this study and will be discussed later.

### 2.5 GC-EI-MS/MS Method

Derivatized samples were analyzed on two different GC-MS instruments (Table 1). The methods had to be slightly adapted for the specific instruments (different autosampler, syringe volume, dean switch, SSL/PTV, mass sensitive detectors (MSD)/FID, and purge flows). However, the same GC-MS column was used, an MS-compatible 5% diphenyl/95% dimethyl polysiloxane column (Thermo Fisher Scientific; Waltham, MA, USA). To prevent syringe clogging when working with field samples, the autosampler washing procedure was adapted, increasing the washing cycles before and after injection, and choosing *n*-hexane/DCM (1:1) as the washing solvent.

**Table 1:** Overview of Instrument Specificities

	setup A	setup B
**instrument**	Shimadzu GC2010 (Kyoto, Japan)	Thermo Trace GC Ultra (Thermo Fisher, Bremen, Germany)
**injector**	Split/splitless (SSL) injector	Programmable temperature vaporizer (PTV)
**column parameters**	TG5MS (Thermo Fisher Scientific; Waltham, MA, USA)	
	length: 30 m length	
	internal diameter: 0.25 mm	
	stationary phase thickness: 0.25 μm	
**liner**	deactivated packed wool glass SSL inlet liner (Shimadzu, Sydney, Australia)	straight deactivated PTV metal liner (Thermo Fisher Scientific; Waltham, MA, USA)
**injection parameters**	injection volume: 1 μL	
	temperature: 300 °C	
	split ratio: 1:25	
	carrier gas: helium	
**flow programming**	mode: flow control	
	carrier gas flow rate: 2 mL/min	
**oven temperature programming**	60 °C (2 min), 10 °C/min to 300 °C (9 min)	
	total run time: 34 min	
**detectors**	TQ8040 MS (triple quadrupole)	flame ionization detector (FID), TSQ Quantum XLS MS (triple quadrupole)
**FID parameters**		temperature: 300 °C
		airflow: 350 mL/min
		H_2_-flow: 35 mL/min
		makeup (N_2_) flow: 30 mL/min
**MSD parameters**	transfer line temperature: 250 °C	
	electron ionization (EI) source temperature: 200 °C	
	electron energy: 70 eV	
	emission current: 60 μA	
	filament power switch time: 0 min off, 6 min on	
	monitoring ions:	
	1. Full scan (*m*/*z* 40 to 500); scan time: 0.3 s	
	2. Multiple reaction monitoring (MRM, parameters see Figure 1);	
	collision gas: Argon	
**data acquisition and evaluation software**	GCMSsolution version 4.52 (Shimadzu)	Thermo Xcalibur 4.4.16.14 (Thermo Fisher)

**Figure 1:**
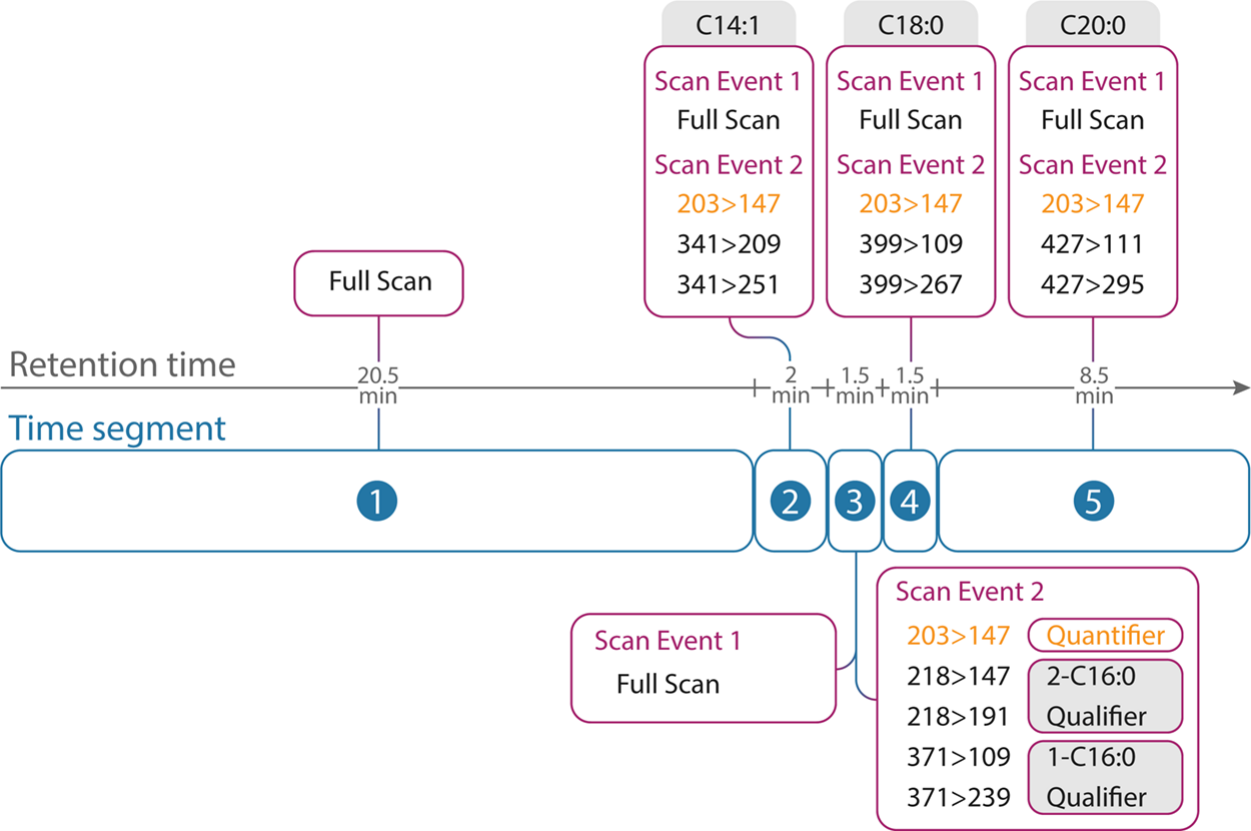
Scheme of time segments and scan events of C14:1, 1-C16:0, 2-C16:0, C18:0, and C20:0 showing full and MRM scans for the improved GC-MS/MS method. Ion transitions are displayed as *m*/*z* values, and quantifier ion transitions are highlighted in orange.

Selected/multiple reaction monitoring (SRM/MRM) was used for quantitative determination, using the third quadrupole as a mass analyzer for molecular identification. The following time segments were implemented: 0 to 20.5 min full scan, 20.5 to 22.5 min—IS qualifier and quantifier ions, 22.5 to 24 min C16:0, 24 to 25.5 min C18:0, and 25.5 to 34 min C20:0 analysis (Figure 1). Analytes were scanned in individual time segments, except for 1-C16:0 and 2-C16:0 due to their very similar retention times. For every analyte, a full and a MRM scan was conducted. The full scan was run at an event time of 0.3 s, and the MRM scans (selective for specific analyte) had a scan event time of 0.2 s. The MRM events are listed in Figure 1; the MRM transition from *m*/*z* 203 > 147 was selected as quantifier ions for all analytes, and the chosen qualifier ion MRM transitions of the specific analytes are stated in the figure. Qualifier ions are those specific to the analyte; quantifier ions are not specific to the analyte but show the highest intensities. Only when the qualifier response was sufficient, the quantifier response was evaluated. Analyte response was normalized to the IS. Product ions and collision energies (CE) were optimized for each analyte. The determined CE giving the best overall ion intensities was 6 V for the IS and 9 V for the SMG references. Structures were identified based on a similarity search against NIST11 and NIST20 libraries and compared to reference substances for 1-monopalmitin, 2-monopalmitin, monostearin, and monoarachidin.

Reference materials were evaluated according to their long-term stability and discrimination effects within the multicomponent (single components vs multicomponent mix injections). LODs and LOQs were determined in CHCl_3_ and CHCl_3_ + 4 wt % B0. The LODs and LOQs were defined as the mean out of nine blank measurements (solvent) plus three times (LOD) or nine times (LOQ) the standard deviation of the blank. Details are available in 2.3 long-term stability tests, 2.4 single standard injections for discrimination effect study, and 2.6.2 LOD/LOQ determination.

### 2.6 HR-ESI-MS Experiments

Fuel samples and reference materials were analyzed qualitatively with HR-ESI-MS for contamination and purity. B7 samples and reference materials were prepared as 1:100 volumetric dilutions in a CHCl_3_:MeOH solvent mixture (v:v, 7:3). Diesel B0 was investigated for SMG contamination in a volumetric 1:100 dilution using MeOH. The samples were injected in an LTQ Orbitrap XL hybrid tandem HR-MS (Thermo Fisher; Waltham, MA, USA) system via direct infusion at 5 μL/min and measured in positive and negative ion modes. Spectra were recorded from *m*/*z* 50 to 800 for precursor ions and from *m*/*z* 60 to 800 for product ions, with a resolution of 30,000. Within the ESI source, nitrogen was used as the sheath and drying gas. Helium served for cooling and as a collision gas during low-energy collision-induced dissociation (CID). Data processing and interpretation were conducted with Thermo Xcalibur 4.4.16.14, Thermo FreeStyle 1.8 SP2, and Mass Frontier 8.0 SR1 (all from Thermo Fisher Scientific Inc.).

## 3 Results and Discussion

### 3.1 Calibration and Sample Solvent

The SMG standard samples showed the same solubility behavior in DCM and CHCl_3_. Due to the easier handling of CHCl_3_ compared to DCM concerning vapor pressure and vapor toxicity, CHCl_3_ was chosen over DCM. Additionally, less solvent evaporation during handling improves the reproducibility of the sample preparation and makes it more independent of the temperature. It must be mentioned that both solvents are considered cancerogenic, and substitution is advised for a greener analysis approach. Furthermore, gloves that are compliant with a higher standard (EN 374^23^) than common nitrile gloves are required for DCM handling due to short breakthrough times.^24^

### 3.2 MRM Optimizations

#### 3.2.1 Collision Energy (CE)

**Figure 2:**
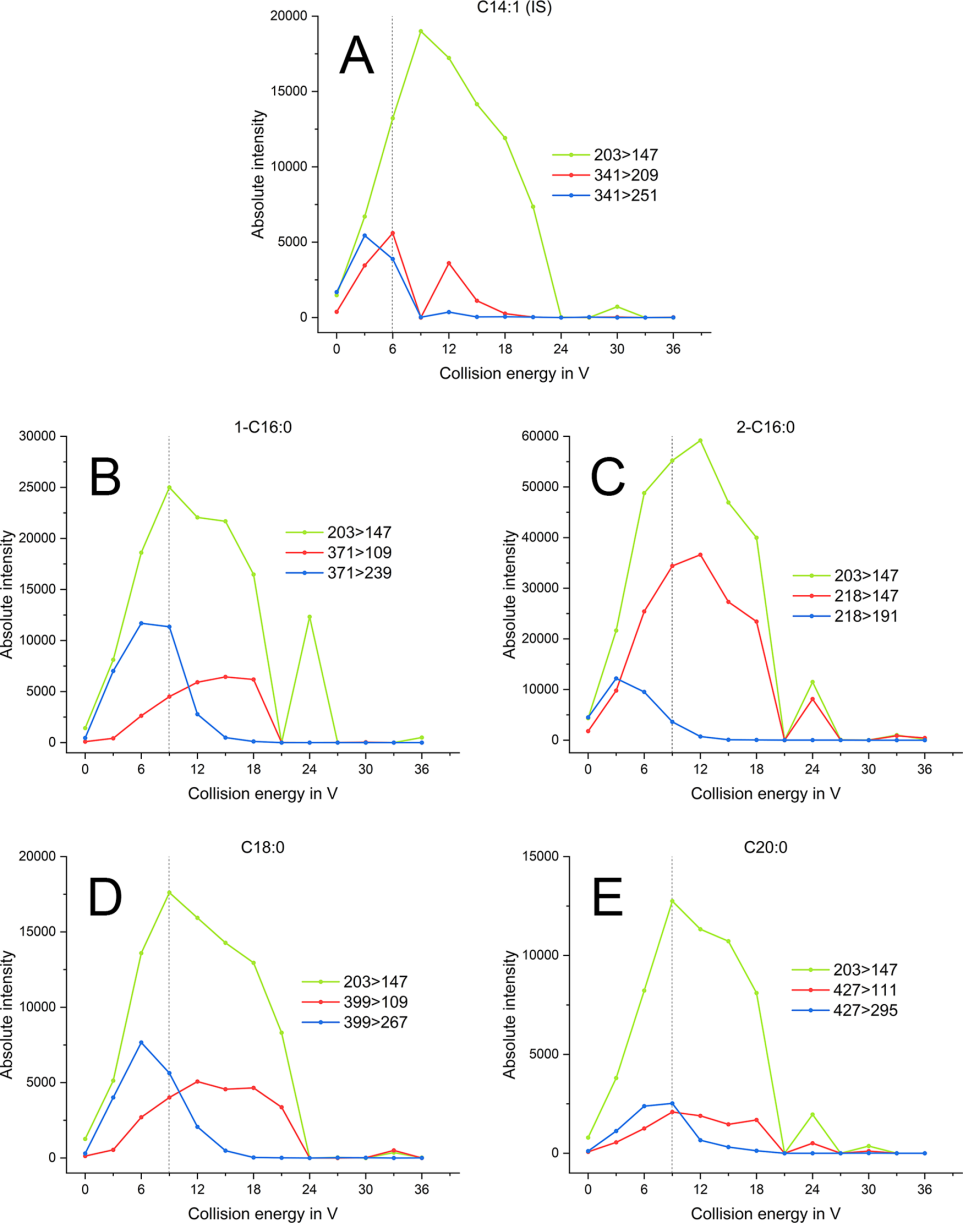
Intensities of the specific qualifier (blue and red lines) and quantifier ions (*m*/*z* 203 > 147, green lines) at different collision energies are shown for the IS C14:1 (A), the single SMG standards 1-C16:0 (B), 2-C16:0 (C), C18:0 (D), and C20:0 (E). Dotted line marks the chosen collision energy at 6 V (A) or 9 V (B–E).

A variation of CEs from 0 to 36 V (in 3 V steps) was tested using a 100 ppm mix with CHCl_3_ plus 4 wt % B0 for every qualifier and quantifier ion of the selected SMG analytes. The CE with the best response of the quantifier ion in combination with a reasonable response for both qualifier ions was selected. The second most abundant ion (*m*/*z* 203) was picked over the most abundant ion (*m*/*z* 147) since the latter was present in the fuel matrix at the same retention time as SMGs, negatively impacting the signals by overlapping. A CE of 6 V was chosen for the IS, and for the SMG analytes, 9 V CE was selected (Figure 2). For 2-C16:0, 12 V CE would have given the best results concerning the quantifier ion, but since the scan was performed in the same time segment as 1-C16:0, due to both substances eluting within a few seconds apart, 9 V was chosen as a good compromise.^25^

#### 3.2.2 Scan Time and Mass Scans

Before adaptation of the method, the peaks of the quantifier ions were evaluated in SIM mode. Typically, a default of one s is used as scan time, which is the time it takes to accomplish one scan. Multiple qualifier and quantifier ions were scanned simultaneously for only 0.2 s to improve data collection, which directly impacted peak shape quality and the number of data points per peak. The full scan time was set to 0.3 s. The scan range was reduced from *m*/*z* 40 to 650 to *m*/*z* 40 to 500 for an additional sensitivity increase.

The number of mass scans within a scan event influences data points over time and by this peak shape and data quality. The fewer *m*/*z* values scanned per event, the more data points available per period. Therefore, the process was limited to a maximum of five masses per scan event. Furthermore, sensitivity could be considerably increased by introducing the MRM measurement in time segments.

### 3.3 Long-Term Stability Tests

**Figure 3:**
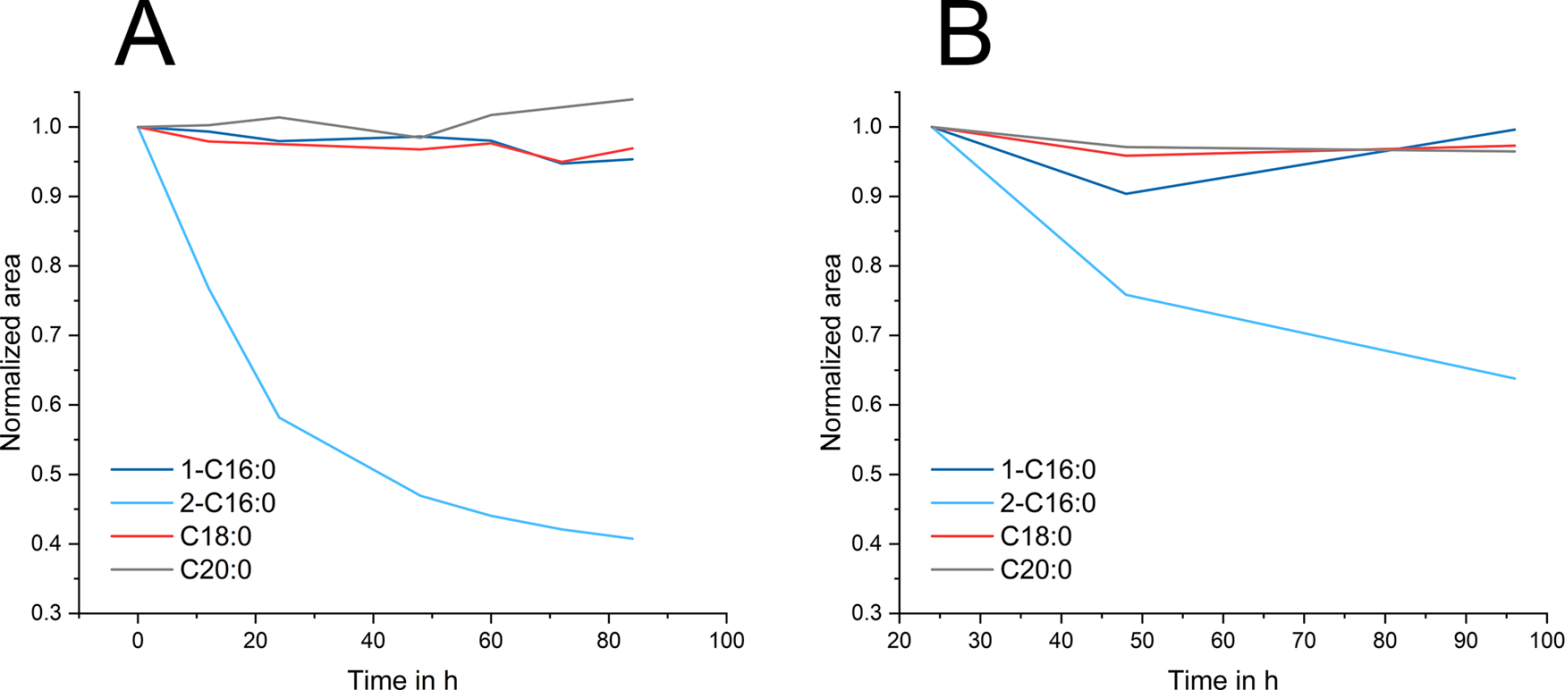
Stability of standards over (A) 82 h, sampled from the same vial, and (B) over 92 h, sampled from fresh vials to overcome solvent evaporation. Data are normalized against IS.

The stability of the multicomponent mix was tested in CHCl_3_ + 4% B0 over a time period of 84 h. Solvent evaporation plays the most crucial role, which is corrected by the IS. Despite a punched vial septum, there was no decrease of area ratios observed for 1-C16:0, C18:0, and C20:0 after normalization with the IS, displaying this good stability over time. Only 2–16:0 decreases by 23% within the first 12 h, which increases to 59% signal area loss after 84 h. When sampling out of fresh, unpunched vials, there is a loss of about 36% between 24 and 96 h for 2-C16:0 (Figure 3). All other standards have more than 95% area recovery. The biggest loss of the 2–16:0 signal is expected to be within the first 20 h. The only notable structural difference between 2-C16:0 and the other SMGs is the linkage of the fatty acid to a secondary alcohol instead of a primary alcohol. This is apparently the main reason for this effect. These experiments were conducted just once to illustrate the observed deviation from other results, which affect the handling of the different SMGs. As for now, this effect was not primarily the objective of the study and, therefore, was not further investigated. The results show that a fresh preparation of the sample and immediate measurement are necessary for reliable collection of data, especially for 2-C16:0.

### 3.4 Single Standard Injections for Discrimination Effect Study

Single standards and the multicomponent mix were compared for their performance with CHCl_3_ and CHCl_3_ + 4% B0. For the normalized (IS and wt %) single standards, the recovery of signals in CHCl_3_ (normalized to 100%) was slightly better than CHCl_3_ + 4% B0, where a loss of up to 9% was observed. The same is valid for the multicomponent mix, where recovery of signals for CHCl_3_ + 4% B0 is approximately 15–17% lower than in pure CHCl_3_ (Table 2). Again, this aligns with the above results, confirming that CHCl_3_ without B0 is the better solvent option. The area recovery is better for the single standards than the multistandard mix (discrimination effect). Still, a normalization by the IS area antagonized this effect. Furthermore, minor concentrations of SMGs were detected in B0 samples, where at first a carryover within GC-MS measurements was expected but was later confirmed by HR-ESI-MS (see discussion in Section 3.5 HR-ESI-MS of standards for contamination evaluation).

**Table 2:** Recovery of Areas of CHCl_3_+4% B0 Compared to Neat CHCl_3_ (Normalized to 100%) Showing IS and wt % Normalized Areas

**standard**	**4% B0/CHCl**_**3**_**recovery**	
	**multi, %**	**single, %**
1-C16:0	83	99
2-C16:0	83	95
C18:0	84	91
C20:0	85	100

### 3.5 HR-ESI-MS of Standards for Contamination Evaluation

With HR-ESI-MS, the neat reference materials for SMGs 1-C16:0, 2-C16:0, C18:0, C20:0, IS C14:1, and B0 were analyzed for their purity and possible contamination. No contamination was found in the MG reference standard samples. Unexpectedly, the B0 sample showed minor impurities of C16:0 and C18:0, which are possibly due to using the same fuel trucks for B0 and B7 when delivered to the fuel station (fuel tanks at the station are always filled with the same type for all tested fuels in this study).

### 3.6 Application of the Method

#### 3.6.1 Calibration

The calibrations of neat CHCl_3_ and 4 wt % B0 in CHCl_3_ were compared. In general, neat CHCl_3_ showed better response ratios for all the analytes and had the advantage of a more independent sample preparation procedure (no diesel sample involved, no additional contamination introduction with the diesel sample) and fewer pipetting steps.

**Figure 4:**
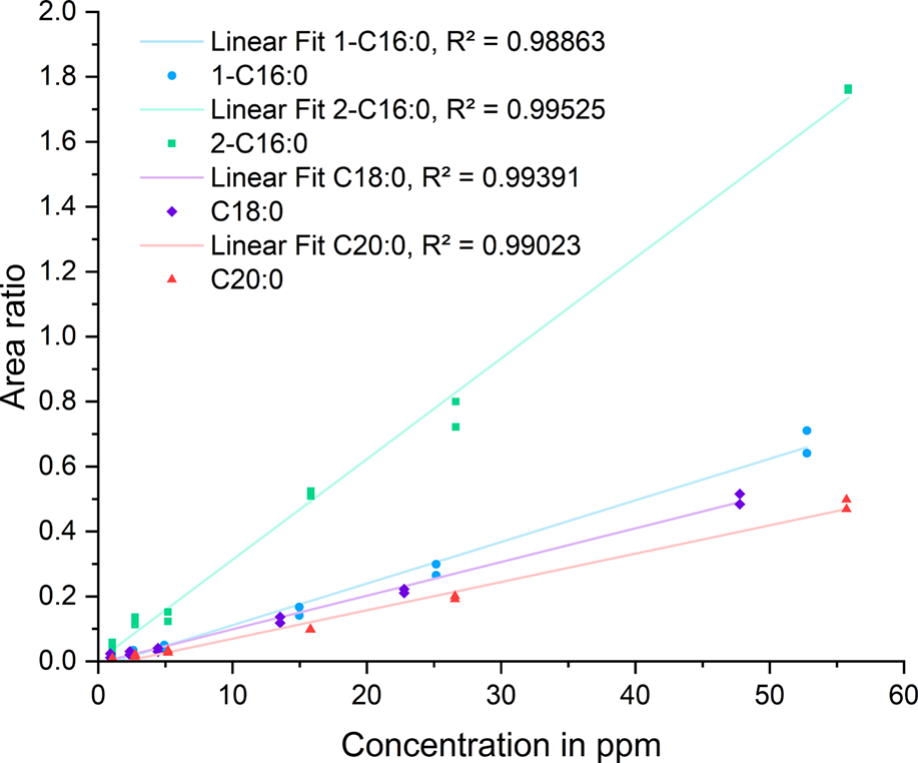
Calibration functions for 1-C16:0 (light blue), 2-C16:0 (green), C18:0 (violet), and C20:0 (red) at different concentration levels in CHCl_3_.

The expected concentration of total SMGs in prepared B7 field samples usually lay within 4 to 15 ppm. Therefore, the calibration curve was focused on the lower concentration levels and carefully evaluated between 1 and 50 ppm (six levels) instead of 10 to 1000 ppm (Figure 4). The sensitivity of the calibration function (slope) strongly affects the LOQ. All calibration curves showed a good fit above 98.8% (*R*^2^ coefficient of determination) (Table 3). The more sensitive measurements for 2-C16:0 again show different behavior from the other MGs.

**Table 3:** Values of Linear Fit Evaluation for the Four Different SMG Calibration Curves

	**1-C**16:0	**2-C**16:0	**C**18:0	**C**20:0
**equation**	*y* = *a* + *b* × *x*	*y* = *a* + *b* × *x*	*y* = *a* + *b* × *x*	*y* = *a* + *b* × *x*
**intercept**	–0.017 ± 0.011	0.003 ± 0.018	–0.005 ± 0.006	–0.017 ± 0.007
**slope**	0.013 ± 4.344 × 10^–4^	0.031 ± 6.770 × 10^–4^	0.010 ± 2.568 × 10^–4^	0.009 ± 2.741 × 10^–4^
**residual sum of squares**	0.007	0.020	0.002	0.003
**Pearson’s *r***	0.994	0.998	0.997	0.995
***R*-square (COD)**	0.989	0.995	0.994	0.990

#### 3.6.2 LOD/LOQ Determination

Blank samples were prepared as three individual sample preparations with three injections each (3 × 3; nine injections total). The area at the position of the quantifier ion was integrated for all analytes, giving blank areas (Table 4). The LODs and LOQs were determined in neat CHCl3, allowing the detection and quantification of SMGs to very low levels.

**Table 4:** LODs and LOQs of Blank Samples of CHCl_3_

**vial**	**concentration in ppm**			
	**1-C**16:0	**2-C**16:0	**C**18:0	**C**20:0
**LOD**	≤2.2	≤1^a^	≤1.3	≤2.0
**LOQ**	≤3.4	≤1^a^	≤2.4	≤2.1

a LODs and LOQs of 2-C16:0 were below the lowest calibration level and corrected to the concentration of the lowest calibration level.

The following LODs and LOQs could be established:

#### 3.6.3 Field Samples

Water was determined in the B7 diesel samples (*n* = 37) with an average of 33 ± 11 ppm. The first and third quartiles are 24 and 43 ppm, giving an interquartile range of 19 ppm. The maximum or fourth quartile was defined as 51 ppm. All the values were lying below the maximum water content allowed for diesel (B0, B7), which is limited to 200 ppm by EN 590.^2^ No direct influence of the water content on the filter blocking ability of the fuel was determined. This was already investigated in a prior study;^15^ for the analyzed samples, no influence of water was revealed.

Twenty-eight different B7 samples were measured, providing the following data (Table 5): the total SMG content was mainly determined by 1-C16:0 and C18:0. The majority of the B7 samples are to be expected within a range of 97 to 142 ppm. The lowest total SMG concentration was 64 ppm, and the highest was 189 ppm; this can be calculated to be approximately 900 to 3200 ppm in B100 (with 7000 ppm being the upper limit for total SMGs in biodiesel by DIN EN 14214^16^). 2-C16:0 and C20:0 concentrations were below the LOQ. A decrease in SMG concentration from the time of sample preparation until their measurement has to be considered (e.g., precipitation because of crystallization), especially for 2-C16:0. Before establishing the method for routine analysis, this observation has to be further investigated and adapted if needed since a fresh preparation of samples for analysis is not always feasible.

**Table 5:** Statistical Evaluation of the Total SMG Concentrations above the LOQ in 28 Different B7 Field Samples

**sample number**	28
	**concentration in ppm**
**mean**	119.72
**minimum**	63.76
**1st quartile**	96.86
**median**	119.11
**3rd quartile**	141.80
**interquartile range**	44.94
**maximum**	188.89

For completeness, neat B0 (pure diesel) and B100 (pure biodiesel) samples were measured. The B0 sample exhibited 75 ppm of SMGs, consisting of 73 ppm of 1-C16:0 and 2 ppm of 2-C16:0, with traces of C18:0 and C20:0 below the LOQ. The 1-C16:0 value seems unusually high for a common B0 diesel, especially since it is above the median value of 72 ppm of B7. Contamination of the B0 sample was expected and confirmed by HR-ESI-MS (see Section 3.5 of HR-ESI-MS of Standards for Contamination Evaluation). For B100, a total concentration of SMGs of 771 ppm was detected; 393 ppm 1-C16:0, 25 ppm 2-C16:0, 296 ppm of C18:0, and 57 ppm of C20:0 were determined.

Regarding filter blocking, 25 differently behaved B7 samples were compared. Nonblocking samples were observed to have an interquartile SMG range from 101 to 137 ppm (median 119 ppm), partly overlapping with blocking samples within a range of 120 to 152 ppm (median 143 ppm). A differentiation was clear for blocked filters, where a high concentration of SMGs was found, whereas in nonblocked filters, mainly unsaturated MGs were discovered.^15^ The blocking of a filter is caused by the accumulation of SMGs on the filter surface over time, which is dependent on the concentration of SMGs in the fuel but also on the rate of flow through the filter, resulting in a faster blocking when the concentration and/or flow rate are high.

## 4 Conclusions

Fuel manufacturers and distributors expressed the need for a precise, reliable, and efficient method to assess the filter blocking potential of diesel, biodiesel, and blends. Additionally, the demand for the ability to differentiate between high-quality and poor-quality fuels served as a driving force for the hereby established GC-EI-MS/MS method.

For improved method performance, various changes to the standard method were introduced: (a) changing the sample solvent from DCM to CHCl_3_ improved the overall reproducibility of sample preparation, (b) changing the solvent system from a matrix-matched calibration standard (diesel plus solvent mixture) to neat solvent (CHCl_3_) not only improved area recovery (only 83–85% area recovery of CHCl_3_+B0 to neat CHCl_3_ in the multicomponent standard) but also made the whole method independent from resources and better for comparability of results between different laboratories, and (c) implementing MRM with optimized collision energies and time segments into the GC-MS method resulted in a highly increased overall sensitivity for all four tested SMGs (1-C16:0, 2-C16:0, C18:0, and C20:0). For the internal standard C14:1, a CE of 9 V would have given the best result for the quantifier ions, but the two qualifier ions were not detected. Hence, 6 V was chosen as the best option for all three ions. CEs of 6–12 V for 1-C16:0 and C18:0 and 6–9 V for 2-C16:0 and C20:0 allow good detection of quantifier and qualifier ions. Scan times of 0.2 s for MRM and 0.3 s for full scan analysis gave satisfactory peak shapes. Surprisingly, the results of HR-ESI-MS showed trace impurities of C16:0 and C18:0 in neat B0 diesel.

The improved GC-MS/MS method allowed the detection of single SMG concentrations down to a minimum concentration (LOD) of 2.2 ppm and quantification (LOQ) down to at least 3.4 ppm in the GC vial, which corresponds to concentrations of 55 (LOD) and 85 ppm (LOQ) in the neat B7 sample. The total SMG concentration in B7 fuel was detectable and is expected to be between 60 and 190 ppm. B7 samples that cause filter blocking are slightly higher in their total SMG content (median of 143 ppm) than samples that do not cause filter blocking (median of 119 ppm). The water content of the fuel samples did not influence the filter blocking ability of the fuel. Still, a more extensive field sample study may be necessary for a valid comparison, including the effects leading to the accumulation of SMGs on filter surfaces over time and a comparison of the SMG concentrations accumulated on the filter and measured in the corresponding fuel for a more comprehensive picture. When comparing SMG amounts of fuels from different stations or on filters and in their related fuels, the samples might be drawn from various levels within the tank due to diverse tank geometries, which will influence the amount of precipitate collected and the resulting measured SMG concentrations, causing fluctuations when comparing nonblocked and blocked filter fuels.

The improved GC-MS/MS method generally led to a more sensitive detection of SMGs in the B0, B7, and B100 matrices. It can be used as an additional quality control for the purchase of biodiesel blend components and premixed diesel blends, for random testing, and in the event of filter blockage, as an accurate assessment based solely on current standards is insufficient. If stricter regulations for the total and single SMG amounts are to be implemented, this method may serve as a standard operating procedure. A fully automated data evaluation can also be implemented, depending on the instrument and data processing software.

A significant loss of signal of normalized 2-C16:0 over time (already within the first 12 h) was registered during long-term stability tests, while other normalized SMGs were stable over time. Various reasons can be identified for this observation: precipitation of the SMG in the original sample or sticking of 2-C16:0 to the sample container walls. More significant losses in sensitivity can still be compensated by using the peak area ratio, giving accurate results. This finding underscores the importance of fresh sample preparation and analysis.
